# Constant rate of p53 tetramerization in response to DNA damage controls the p53 response

**DOI:** 10.15252/msb.20145168

**Published:** 2014-10-24

**Authors:** Giorgio Gaglia, Galit Lahav

**Affiliations:** Department of Systems Biology, Harvard Medical SchoolBoston, MA, USA

**Keywords:** DNA damage, fluorescence imaging, p53 dynamics, single cells, tetramers

## Abstract

The dynamics of the tumor suppressor protein p53 have been previously investigated in single cells using fluorescently tagged p53. Such approach reports on the total abundance of p53 but does not provide a measure for functional p53. We used fluorescent protein-fragment complementation assay (PCA) to quantify in single cells the dynamics of p53 tetramers, the functional units of p53. We found that while total p53 increases proportionally to the input strength, p53 tetramers are formed in cells at a constant rate. This breaks the linear input–output relation and dampens the p53 response. Disruption of the p53-binding protein ARC led to a dose-dependent rate of tetramers formation, resulting in enhanced tetramerization and induction of p53 target genes. Our work suggests that constraining the p53 response in face of variable inputs may protect cells from committing to terminal outcomes and highlights the importance of quantifying the active form of signaling molecules in single cells.

Quantification of the dynamics of p53 tetramers in single cells using a fluorescent protein-fragment complementation assay reveals that, while total p53 increases proportionally to the DNA damage strength, p53 tetramers are formed at a constant rate.

## Introduction

**Figure 1:**
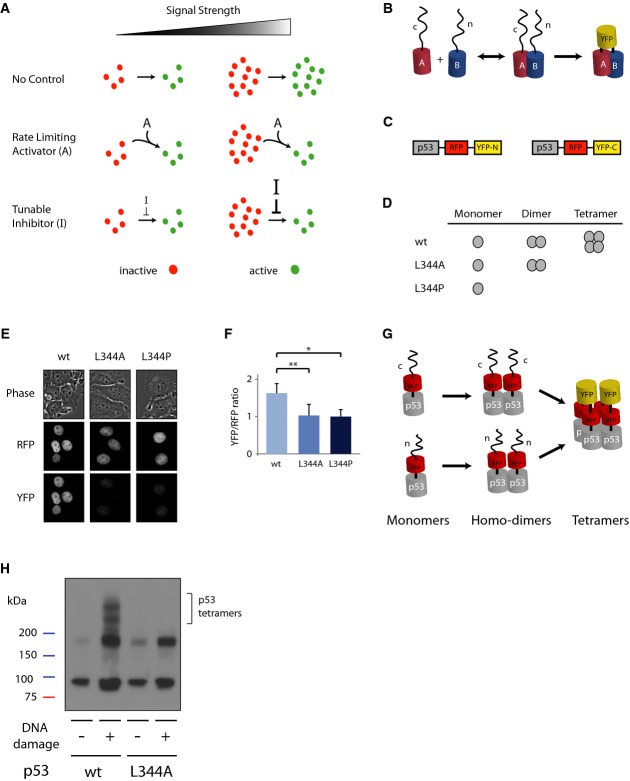
The dynamics of p53 tetramerization in cells can be studied using Venus yellow fluorescence protein-fragment complementation assay (vYFP PCA) Schematic drawing of potential mechanisms constraining the levels of an active molecule. In all cases, the levels of an inactive molecule (red) are proportional to the signal strength. In the absence of a control system (top), the levels of the active form (green) are also proportional to the signal strength. The linear relationship between signal strength and molecule activation can be broken by the presence of a constant activator “A”, which limits the rate of activation (middle), or by the presence of a tunable inhibitor “I”, the strength of which depends on the strength of the signal (bottom).PCA is based on tagging putative interactive proteins (“A” and “B”) with two different fragments of a fluorescence protein (“n” and “c”). The interaction between A and B brings the unfolded non-fluorescent fragments in close proximity and they form a full fluorescent protein.Schematic drawing of the p53 reporters.p53 species; p53 L344A forms dimers but not tetramers, while p53 L344P is only monomeric.Images of cells expressing the constructs in (C) with mutant or wild-type p53s in bright-field illumination, RFP and YFP.Ratio of YFP to RFP fluorescence level in cells. Median and standard deviation are reported, and values are normalized to p53 L344P (*n* ≥ 50, *P = 10^−17^, **P = 10^−14^, p53 L344A and p53 L344P are not statistically significantly different *P* = 0.22; *P*-values obtained by Mann–Whitney *U*-test).p53 forms homo-dimers, in which both monomers are tagged with the same fragment of YFP leading to no YFP signal. When dimers form tetramers the split YFP protein is formed and becomes fluorescent.Lysate crosslinking with 0.025% glutaraldehyde with or without DNA damage induction by NCS (400 ng/mL) on p53 wild-type and p53 L344A dimeric mutant tagged with YFP PCA reporter system (C).

Biological systems often exhibit a graded response in which the stronger the input, the higher and broader the output. However, in some systems, this simple relationship is constrained, buffering against fluctuations and extreme signals or deferring the response (Alon, 2007; Mettetal *et al*, 2008; Levine *et al*, 2012; Kim *et al*, 2013). Restriction of the output can be achieved, for example, by a rate-limiting activator not affected by the input (“A” in Fig1A). Alternatively, constant activation in face of variable input strengths can result from an inhibitory mechanism. In this scenario, the activation is constrained by a tunable valve, the function of which increases with the input strength (“I” in Fig1A). Such a mechanism, referred to as a throttle, is commonly used in engineering. In order to identify and characterize such potential mechanisms in biology, we need to be able to accurately measure both the total level of a signaling protein and its active form in the same cell in response to variable input strength. Here, we quantified the total level of the tumor suppressor p53 and its active tetrameric form in single cells in response to a range of UV doses and identified a throttling mechanism for damping p53 activity at high UV levels.

The p53 protein is induced in response to stress and triggers different cellular outcomes including cell cycle arrest, apoptosis, and senescence (Vogelstein *et al*, 2000). Fluorescence reporters of p53 have previously been used to study the dynamics of p53 in live cells (Lahav *et al*, 2004; Batchelor *et al*, 2008; Loewer *et al*, 2010). These studies revealed that p53 dynamics depend on the stimulus and affect cellular outcomes (Purvis *et al*, 2012). UV radiation, for example, leads to a transient increase in p53 protein level displaying a single-graded pulse. The amplitude and duration of the pulse depend on the UV dose, with higher doses leading to stronger and longer p53 induction (Batchelor *et al*, 2011) and to cell death (Purvis *et al*, 2012). However, fluorescently tagged p53 reports only for the dynamics of *total* p53 and does not capture the dynamics of *active* p53, which depends on specific modifications and homo-oligomerization.

Activity of transcription factors in single cells can be quantified using various methods. In cases where the transcription factor is regulated through localization, fluorescent tagging was used to report for transcriptional activity (Cai *et al*, 2008; Hao & O'Shea, 2012). In many cells, p53 is stably localized in the nucleus, and therefore, localization is not a sufficient measure for its activity. Transcription factors' activity in cells can also be measured by a transcriptional reporter, in which a target gene promoter drives the expression of a fluorescent protein. Such an approach has been used, for example, to study the activity of the circadian clock gene Per1 (Quintero *et al*, 2003). In the p53 pathway, different target genes show different patterns of activation, implying that their induction depends on additional factors beyond p53 and making it impossible to choose a single promoter as a general readout for p53 activity (Purvis *et al*, 2012).

Tetramerization of p53 has been shown to be fundamental for its ability to bind DNA and activate transcription, suggesting tetramerization as a valuable measure for globally quantifying the functional unit of p53 in single cells. Mutations in the p53 tetramerization domain (326–356 aa) lead to a reduction in, or loss of, its transcriptional activity in cells (Kawaguchi *et al*, 2005) and were shown to cause early cancer onset, known as Li–Fraumeni syndrome (Davison *et al*, 1998; DiGiammarino *et al*, 2002). The formation of p53 tetramers is driven by a C-terminal tetramerization domain. The reaction proceeds in two steps: first, p53 monomers bind into dimers, which then form tetramers. Hence, p53 tetramers are referred to as “dimers of dimers”. Previous *in vitro* work showed that p53 dimerization occurs co-translationally, on the polysome, suggesting that p53 dimers are composed of monomers translated from the same mRNA (Nicholls *et al*, 2002). Tetramerization occurs post-translationally and is regulated by specific post-translational modifications and co-factor binding (Foo *et al*, 2007; Rajagopalan *et al*, 2008; Schumacher *et al*, 2010).

We recently used fluorescence correlation spectroscopy (FCS) to measure the tetramerization of p53 in single cells (Gaglia *et al*, 2013). This method allowed quantifying the stoichiometry of p53 oligomers directly in live cells and monitoring their temporal changes after DNA damage. However, FCS is a low-throughput method; the number of cells measurable by FCS is limited (∼5–20), and the single-cell dynamics can currently only be measured manually. Here, we developed a fluorescent protein-fragment complementation assay (PCA) to quantify the dynamics of p53 tetramers in single cells and investigated how cells regulate total p53 and its activity under variable input strength.

## Results and Discussion

### Fluorescent PCA captures p53 tetramerization in single cells

To investigate the dynamics of p53 oligomerization in cells, we used a Venus yellow fluorescent protein-fragment complementation assay (vYFP PCA) (Remy *et al*, 2004). PCA relies on splitting a fluorescent protein in two complementary fragments, and tagging each to one of two proteins that potentially bind each other. The split fragments are natively unfolded, not fluorescent alone, and bind each other with low affinity. When they are brought together by the stable interaction of the proteins they are tagged to, the protein is able to fold, leading to a stable fluorescent protein (Fig1B) (Ghosh *et al*, 2000; Magliery *et al*, 2005; Michnick *et al*, 2007) which does not disassemble. We tagged two copies of p53 to two different fragments of Venus YFP (YFP-N or YFP-C) and stably expressed them in human cells. Each p53 was also tagged to a full-length red fluorescent protein (RFP) to report for the total p53 protein in cells (Fig1C). In principle, the formation of both p53 dimers and tetramers could yield fluorescence. In order to separate the contribution of each of these states to our measurements, we used two well-characterized mutants of p53: p53 L344A that forms dimers, but not tetramers, and p53 L344P, which is exclusively monomeric (Fig1D). As expected, the p53 L344P monomeric mutant showed low YFP signal (Fig1E), representing auto-fluorescence or unspecific binding between the Venus YFP fragments. Notably, the p53 L344A mutant, which is able to form dimers, did not display higher YFP fluorescence than the monomeric mutant (Fig1E). Accordingly, the ratio between the YFP signal to total p53 (measured by the RFP signal) in the L334A mutant was equivalent to the ratio obtained from the monomeric mutant L334P (Fig1F), suggesting that dimerization of p53 does not add fluorescence signal beyond the background observed by the monomeric p53. We confirmed this result using another p53 dimeric mutant, p53 M340E L344K (Davison *et al*, 2001) (Supplementary Fig S1A). Our data suggest that p53 dimers are homo-dimers; every dimer comprises the same two fragments of Venus YFP (1G). This is in agreement with *in vitro* studies showing that p53 dimers are formed co-translationally, consisting of two monomers translated from the same mRNA (Nicholls *et al*, 2002). Once formed, the dimers' low dissociation rate and the short half-life of p53 keep dimers from exchanging monomers. We further tested the homo-dimerization of p53 by a pull-down assay of cells expressing different p53 species fused to HA or CFP (Supplementary Fig S1B–D). Our results show that HA-tagged wild-type p53 successfully pulls down p53-CFP, while the two dimer mutants (L344A and M340E L344K), which are unable to form tetramers, do not. The very faint band observed with the dimer mutant implies that a small fraction of dimers might consist of hetero-dimers. However, the low intensity of this band, even after a long exposure of the membrane, suggests that this low fraction of hetero-dimers, if it exists, is negligible, and does not yield a fluorescence signal beyond the monomeric background as was shown using the vYFP PCA system (Fig1F and Supplementary Fig S1A). An increase in the YFP/RFP ratio therefore predominantly reports on p53 tetramers (Fig1G).

We further confirmed that the complementary fragments of Venus YFP do not interfere with p53′s ability to form tetramers and do not induce spurious tetramerization (Fig1H). Moreover, the irreversible binding of the Venus YFP fragment could in principle perturb the regulation of p53 protein (Magliery *et al*, 2005). We found that the vYFP PCA reporters do not alter the previously well-characterized pulsatile dynamics of total p53 after double-strand breaks (Lahav *et al*, 2004; Batchelor *et al*, 2008), (Supplementary Fig S2), suggesting that irreversible protein-fragment complementation does not affect p53 regulation and dynamics.

### p53 tetramers are formed at a constant rate independent of input strength

**Figure 2:**
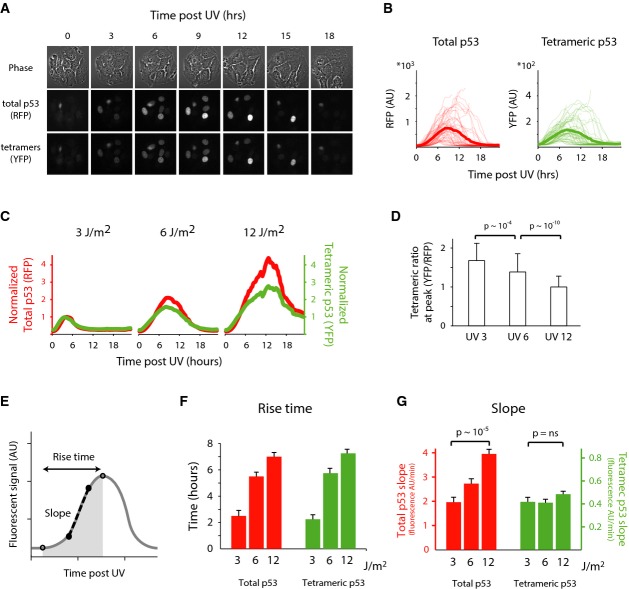
p53 tetramerization is damped at increasing UV doses through a constant rate of tetramers formation A, B Time-lapse images (A) and quantification of total and tetrameric p53 (B) following 6 J/m^2^ UV. Each trace is a single cell. Bold traces represent the mean dynamics (*n* = 100).C Time traces of the mean fluorescent level under three doses of UV irradiation. Red traces represent total p53, and green represent tetrameric p53 levels. Traces were normalized to the respective maximum level of 3 J/m^2^ UV treatment (*n* = 280).D The ratio of tetrameric p53 max levels, attained by YFP, over its max total levels, attained by RFP. Error bars represent standard deviation of the mean. *P* = 10^−4^ and *P* = 10^−10^. *P*-values calculated by Mann–Whitney *U*-test, with *P* = 0.05 as significance threshold.E, F The dynamics of p53 after UV can be captured by two main parameters: the rise time and the slope of increase.Rise time (F) and slope (G) for total and tetrameric p53 at increasing doses of UV. Shown are median and SEM. *n* = 210, *P* = 10^−5^ for total p53 and *P* = 0.09 for tetrameric p53. *P*-values were calculated by Mann–Whitney *U*-test, with *P* = 0.05 as significance threshold. Source data are available online for this figure.

Images of cells expressing the p53 tetramer reporter revealed that UV irradiation triggers a transient single pulse of p53 tetramers similar to the dynamics observed for total p53 (Fig2A and B). However, when we treated cells with a range of UV doses, we observed a major difference between the dynamics of total p53 and tetrameric p53. Higher doses of UV led to an increase in the amount of total p53 as previously reported (Batchelor *et al*, 2011). p53 tetramers also increased with higher UV doses; however, the effect was limited in comparison to total p53 (Fig2C and Supplementary Fig S3A). Quantitatively, the ratio between p53 tetramers and total p53 decreases with higher levels of UV, indicating damping of p53 tetramers (Fig2D and Supplementary Fig S3B).

We next asked what leads to the damping of p53 tetramers in response to high levels of UV. The dynamics of p53 post-UV can be described by two main properties: the rise time (the duration of the increase) and the slope (the rate at which the signal accumulates) (Fig2E and Supplementary Fig S3C). The damping in the ratio between p53 tetramers and total p53 could derive from modulation of either of these two properties. We found that the rise time increases with higher UV doses for both total p53 and p53 tetramers (Fig2F). Damping in p53 tetramers therefore cannot be explained by a difference in rise time. The slopes showed a different behavior; the slopes of total p53 was dose dependent, while the slope of p53 tetramers was constant across UV doses (Fig2G). This explains the damping of p53 tetramers at higher level of UV and suggests a regulatory mechanism maintaining a constant rate of tetramer formation independent of the rate at which total p53 accumulates.

### ARC knockdown breaks the slope conservation and leads to enhanced induction of p53 targets

**Figure 3:**
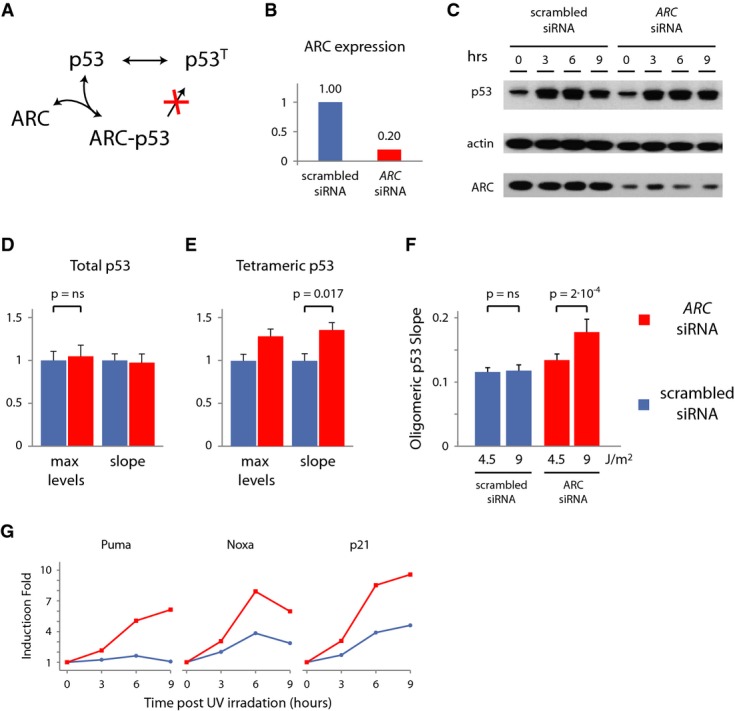
ARC knockdown leads to dose-dependent rate of tetramers formation and enhanced the induction of p53 target expression A The ARC protein binds p53 and interferes with p53 tetramerization.B qPCR of *ARC* mRNA under scrambled and *ARC* siRNA.C Immunoblot of cells following UV at the indicated time points.D, E Maximum levels and slope for total (D) and tetrameric (E) p53. Shown are median and SEM. *n* = 170. ARC knockdown does not affect the total p53 maximum (*P* = 0.62) and slope (*P* = 0.57) but increases the rate of p53 tetramer accumulation (*P* = 0.007 and *P* = 0.017). *P*-values were calculated by Mann–Whitney *U*-test, with *P* = 0.05 as significance threshold.F Knockdown of ARC breaks the slope conservation of tetramers formation. Shown are median and SEM. *n* = 360. *P* = 2 × 10^−4^ for ARC siRNA and *P* = 0.47 for scrambled siRNA control.G Fold induction of p53 target genes quantified by qPCR following 6 J/m^2^ UV after ARC siRNA (red) or scramble siRNA (blue). Source data are available online for this figure.

Maintaining a constant rate of tetramer formation could be achieved by sequestering p53 dimers, preventing them from becoming tetramers, therefore reducing the pool of tetramers' precursors. The apoptosis repressor with caspase recruitment domain (ARC) was previously shown to interact with p53 and disrupt its tetramerization (Fig3A and Foo *et al*, 2007). To test whether ARC is responsible for the fixed rate at which p53 tetramers are formed, we silenced ARC by siRNA (Fig3B and C, and Supplementary Fig S4A) and measured the effect on total p53 and p53 tetramers in single cells. The maximum level and slope of total p53 were not affected by ARC knockdown (Fig3D and Supplementary Fig S4B). Conversely, the dynamics of p53 tetramers were significantly affected by knockdown of ARC; p53 tetramers formed faster, as indicated by the increase in both the slope of tetrameric p53 post-UV and higher maximum level (Fig3E). Moreover, silencing ARC disrupted the slope conservation of tetramer formation across UV doses; higher doses of UV led to steeper slope of p53 tetramers formation (Fig3F), and the damping effect was lost (Supplementary Fig S4C and D).

Does breaking the fixed rate of tetramer formation enhance the transcription of p53 target genes? We measured the induction of p53 target genes after UV treatment with and without ARC. We found that silencing ARC leads to a stronger induction of p53 represented target genes involved in apoptosis and cell cycle arrest (Fig3G) in a p53-dependent manner and to a slight increase in apoptosis (Supplementary Fig S4E and F). This suggests that the increase in the influx of p53 tetramers caused by ARC knockdown boosts p53 activity.

Constant rate of tetramers formation in face of varying UV doses can theoretically be achieved by two distinct mechanisms (Fig1A): (i) a rate-limiting activator of p53 tetramerization, displaying fixed levels and activities independent on the UV dose and (ii) a tunable inhibitor of p53 tetramerization exhibiting stronger inhibition at high levels of UV. Various activators were previously shown to enhance the formation of p53 tetramers, including 14-3-3σ and Hsp70 (Hainaut & Milner, 1992; Rajagopalan *et al*, 2008). While these activators are undoubtedly important for this process, our finding that knockdown of ARC allows p53 tetramers to form faster at higher UV doses (Fig3), indicates that the constant rate of tetramers formation in the p53 systems is achieved through inhibition, and not by a rate-limiting activator (Fig1A).

ARC's inhibitory function creates a molecular throttle, allowing for total p53 protein to accumulate while constraining the formation of p53 tetramers by a tunable valve. The mechanism by which ARC inhibits tetramerization and how the inhibition is tuned in response to various UV doses remain open questions. The fact that ARC protein levels do not change after UV suggests that the regulation of ARC's inhibitory activity requires additional control, such as post-translational modifications or cellular localization (Wang *et al*, 2005). ARC binds directly to the tetramerization domain of p53 (Foo *et al*, 2007) and could potentially compete for the dimer–dimer interaction surface. The fact that the rate of tetramerization is controlled over a wide concentration range of p53, achieved through either the natural increase after UV (Fig2C) or artificially by stabilizing the p53 protein prior to UV induction (Supplementary Fig S5), suggests that ARC abundance in cells is much higher than p53. Alternatively, ARC might act as a mediator only transiently required to disrupt p53 tetramerization, for example, facilitating p53 modifications.

In mechanical engineering, a throttle is often used to regulate the flow of a fluid or gas entering an engine, optimizing a desired property of the engine, such as speed or fuel efficiency. What could be the biological advantages of throttling p53 tetramers formation? p53 triggers crucial outcomes in cells, some are terminal and irreversible. UV, for example, leads to cell death. Executing such outcomes at the right time is an important feature of p53 function. A simple linear relationship between UV dose and p53 levels can be dangerous to cells, as high levels of p53 can activate apoptosis too fast, without allowing cells the time to repair the damage and recover. A fixed rate of tetramers formation creates a “brake” in the formation of functional p53, which may be required for protecting cells from prematurely committing to cell death.

One of the main goals in cancer therapy is to enhance p53 function in cancer cells. Our ability to understand the various constraints on p53 function through modulation of its dynamics or inhibition of its tetramers has important implications for inducing p53 activity in cells. Specifically, our study suggests that enhancing the efficacy of DNA-damaging drugs can be achieved by combining them with drugs that inhibit ARC, breaking the fixed rate of tetramers formations in cells. Other pathways are known to control cell fate decisions in cells. Developing new tools for measuring their activity in single cells can help reveal other potential molecular throttles for properly controlling the balance between alternative cellular outcomes.

## Materials and Methods

### Cell lines

The cell line MCF7+p53shRNA was kindly provided by Reuven Agami group (Brummelkamp *et al*, 2002), the Netherlands Cancer Institute, Amsterdam, the Netherlands. cDNA for p53 was altered by site-directed mutagenesis (QuikChange kit, Stratagene) at residue 344 to obtain oligomerization mutants p53 L344A and p53 L344P, and with 7 silent point mutations that allow for mRNA to escape shRNA silencing without altering the amino acid sequence. p53 was expressed under the EF1α promoter and tagged with the full red fluorescent protein mKate2 and one of the two fragments of mVenus (mVenus-N, 1-158aa and Venus-C, 159-240aa). The vector was introduced in cells via lentiviral infection and stable clonal selection. Lentiviral particles were produced in 293T cells.

### Cell culture and DNA damage

MCF7+p53shRNA+p53-mKate2-mVenus-N/C cells were maintained in RPMI supplemented with 10% fetal calf serum, 100 U/ml penicillin, 100 mg/ml streptomycin, 250 ng/ml fungizone (Gemini Bio-Products) supplemented with selective antibiotics (400 μg/ml G418, 0.5 μg/ml puromycin, 100 μg/ml hygromycin). DNA damage was induced in cells using a UV-C 254-nm light source (Ushio). UV was delivered to cells using a UV lamp with a rate of 1.5 J/m^2^/s. All UV treatments, therefore, were performed in a single burst lasting < 7 s. Cells were harvested for protein/RNA extraction at the indicated times after DNA damage.

### Western blot analysis

Harvested cells were lysed in the presence of protease and phosphatase inhibitors. Total protein levels were quantified using the BCA assay (Pierce). Equal protein amounts were separated by electrophoresis on 4–12% Bis-Tris gradient gels (Invitrogen) and transferred to PVDF membranes by electroblotting. Membranes were blocked with 5% nonfat dried milk, incubated overnight with primary antibody, washed, and incubated with secondary antibody coupled to peroxidase. Protein levels were detected with chemiluminiscence (ECL plus, Amersham). p53 dynamics were quantified by normalizing total p53 levels (DO1; Santa Cruz) to α-actin (Sigma). ARC was probed with a polyclonal antibody from Cayman Chemicals (#160737).

### Target gene expression dynamics

Total RNA was extracted using the RNeasy protocol (Qiagen). RNA concentration was determined by measuring absorbance at 260 nm. Equal RNA levels were used to generate complementary DNA using the high-capacity cDNA reverse transcription protocol (Applied Biosystems). Quantitative PCR was performed using reaction mixtures of 8.4 ng total RNA, 100 nM primer, and SYBR Green reagent (Applied Biosystems).

### Time-lapse microscopy

Two days before microscopy, cells were grown on poly-D-lysine-coated glass-bottom plates (MatTek Corporation) in transparent medium supplemented with 5% fetal calf serum, 100 U/ml penicillin, 100 μg/ml streptomycin, and 250 ng/ml fungizone (Gemini Bio-Products). Cells were imaged with a Nikon Eclipse Ti-inverted fluorescence microscope on which the stage was surrounded by an enclosure to maintain constant temperature, CO_2_ concentration, and humidity. Images were acquired every 15 min. The mVenus filter set was 500/20× excitation, 515 nm dichroic beam splitter, and 535/30 m emission (Chroma). The mKate2 filter set was 560/40× excitation, 585 nm dichroic beam splitter, and 630/75 m emission (Chroma). We analyzed images using MetaMorph software (Molecular Devices) and custom-written MATLAB software (Mathworks), which is available upon request. The peaks and troughs of the fluorescent signal (reporting for total and tetrameric p53) were identified through a watershedding algorithm previously described in Loewer *et al*, 2010. The rise time was defined as the time between the first trough and the first peak. The slope of increase for fluorescent signal (Figs2 and 3) was calculated by computing the maximum difference over a window of 1 h within the timing of the first trough and the first peak. Data and MATLAB codes used to generate Figs2 and 3 are provided as Source Data and described in Supplementary Table S1.

### RNAi

To knockdown ARC, we used siGENOME SMARTpool of siRNA against the NOL3 gene mRNA (Dharmacon). For all controls, we used the scrambled siRNA from Qiagen (AllStars Negative Control siRNA, Qiagen 1027280). We performed all RNA transfection using DharmaFECT 1 transfection reagent following the manufacturer's protocol (Dharmacon). We assayed the knockdown of NOL3 48 h after transfection.

## Supporting Information

Supplementary Information

Source Data for Supplementary Figure S3A

Review Process File

Source Data for Figure 2

Source Data for Figure 3D and E

Source Data for Figure 3F
