# Supplementary material for: Constant rate of p53 tetramerization in response to DNA damage controls the p53 response
Source: Mol Syst Biol. 2014 Oct 24;10(10):1–8. doi: 10.15252/msb.20145168 (PMC4299375; doi:10.15252/msb.20145168)
Supplement: Supplementary file 2 — Source Data for Supplementary Figure S3A [file msb0010-0753-sd2.pdf]

Supplementary Information for:

Constant rate of p53 tetramerization in response to DNA damage controls the p53 response

Giorgio Gaglia[1], and Galit Lahav[1*]

[1]Department of Systems Biology, Harvard Medical School, Boston MA 02115

[*]Corresponding Author:
Galit Lahav
Warren Alpert Building, Room 555
200 Longwood Avenue
Harvard Medical School
Boston, MA 02115
617-432-5621 (phone), 617-432-5012 (fax)
galit@hms.harvard.edu

TABLE OF CONTENTS

Supplementary Figure S1 ….……………………………..…..…………….……………. 3

Supplementary Figure S2 ….……………………………..…..…………….……………. 4

Supplementary Figure S3 ….……………………………..…..…………….……………. 6

Supplementary Figure S4 ….……………………………..…..…………….……………. 7

Supplementary Figure S5 ….……………………………..…..…………….……………. 9

Supplementary Table S1 ….……………………………..…..…………….……………. 10

Supplementary Experimental Procedures ….……………………………..…..……………. 11

Supplementary References ….……………………………..…..…………….……………. 11

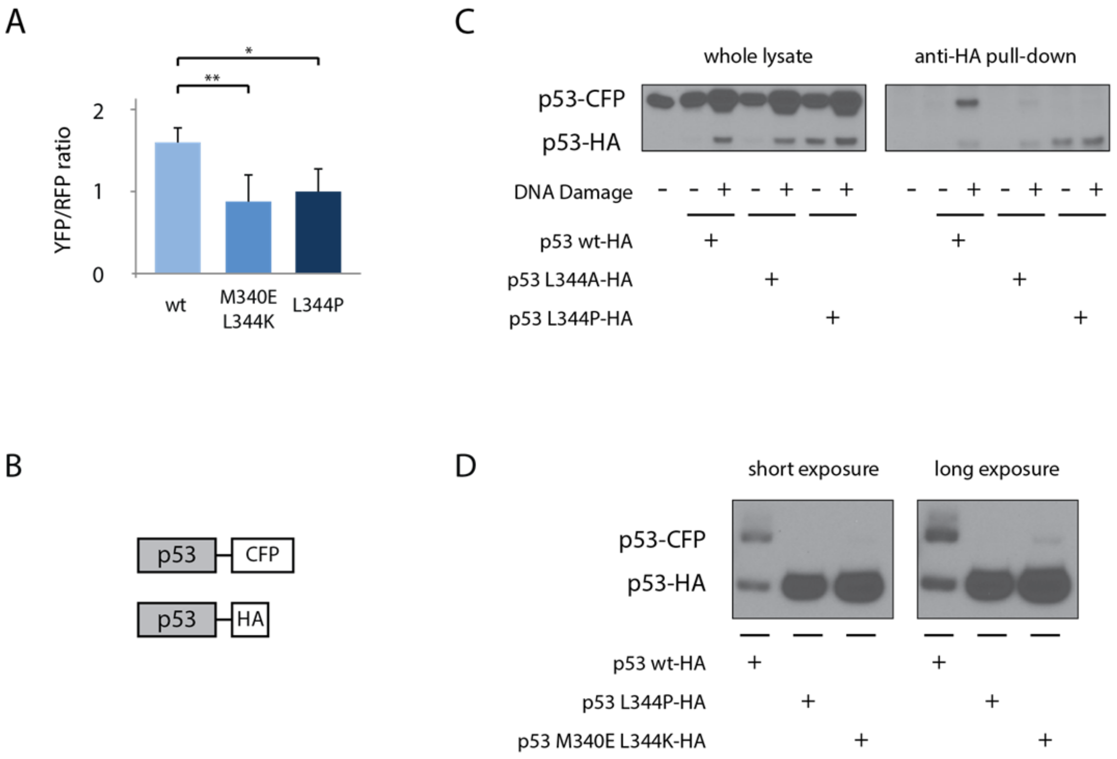

Supplementary Figure S1: Supporting evidence for the efficacy of the vYFP PCA system. (**A**) Ratio of YFP to RFP fluorescence level in cells expressing mutant or wild-type p53. Median and standard deviation are reported and values are normalized to p53 L344P (n • 50, * p ~ 10-12 and ** p ~ 10-15, p-vaules obtained by Mann-Whitney test). (**B**) p53 was tagged with biochemical tags HA and CFP and expressed in cells. (**C, D**) Co-immunoprecipitation experiment in cells expressing wild-type p53-CFP together with either mutant or wild-type p53-HA. Cells were damaged with the radiomimetic drug NCS and lysates were collected 2.5 hours after treatment.

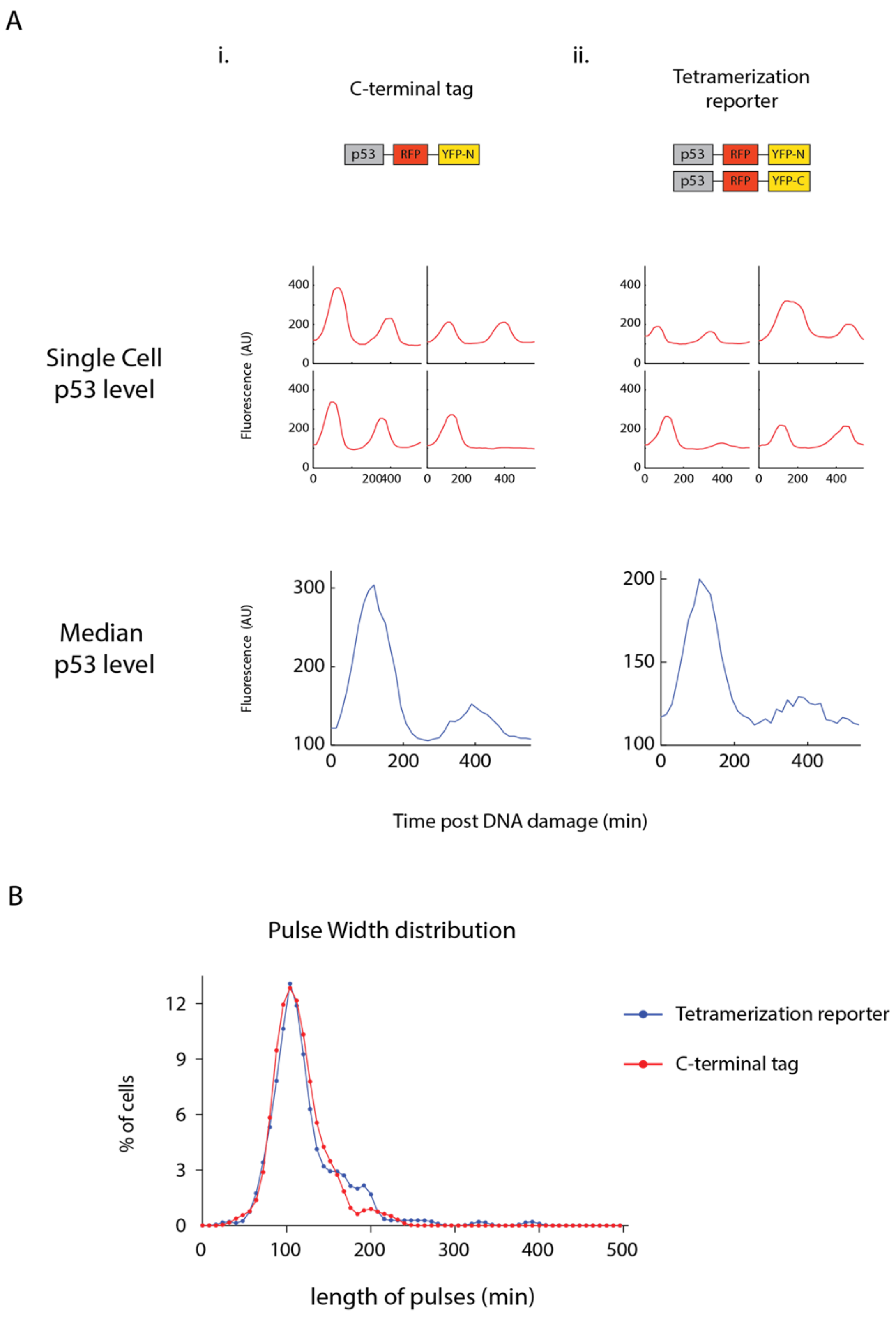
A
i.
C-terminal tag
p53 RFP YFP-N
ii.
Tetramerization reporter
p53 RFP YFP-N
p53 RFP YFP-C
Single Cell p53 level
Fluorescence (AU)
Median p53 level
Fluorescence (AU)
Time post DNA damage (min)
B
Pulse Width distribution
% of cells
length of pulses (min)
Tetramerization reporter
C-terminal tag

Supplementary Figure S2: p53 dynamics after DNA double strand breaks are not altered by the PCA reporter system.

(**A**) MCF7 cells were transformed with either (i) one of the vYFP PCA constructs or (ii) the full vYFP PCA tetramerization reporter system, consisting in two p53 constructs tagged with a full RFP and one of the fragments of split YFP protein. The dynamics of total p53 reported by the RFP signal after double strands breaks induced by NCS (400ng/mL) are presented at single cell level and at median population level (n>40). The presence of the full vYFP PCA system, in which the split YFP is able to bind and reassemble, does not affect the pulsatile dynamics of p53. (**B**) Quantification of the width of total p53 after double strand breaks, measured by the full-width half-maximum. The distributions of peaks are overlapping for the vYFP PCA system and the single construct (p = 0.66, K-S test).

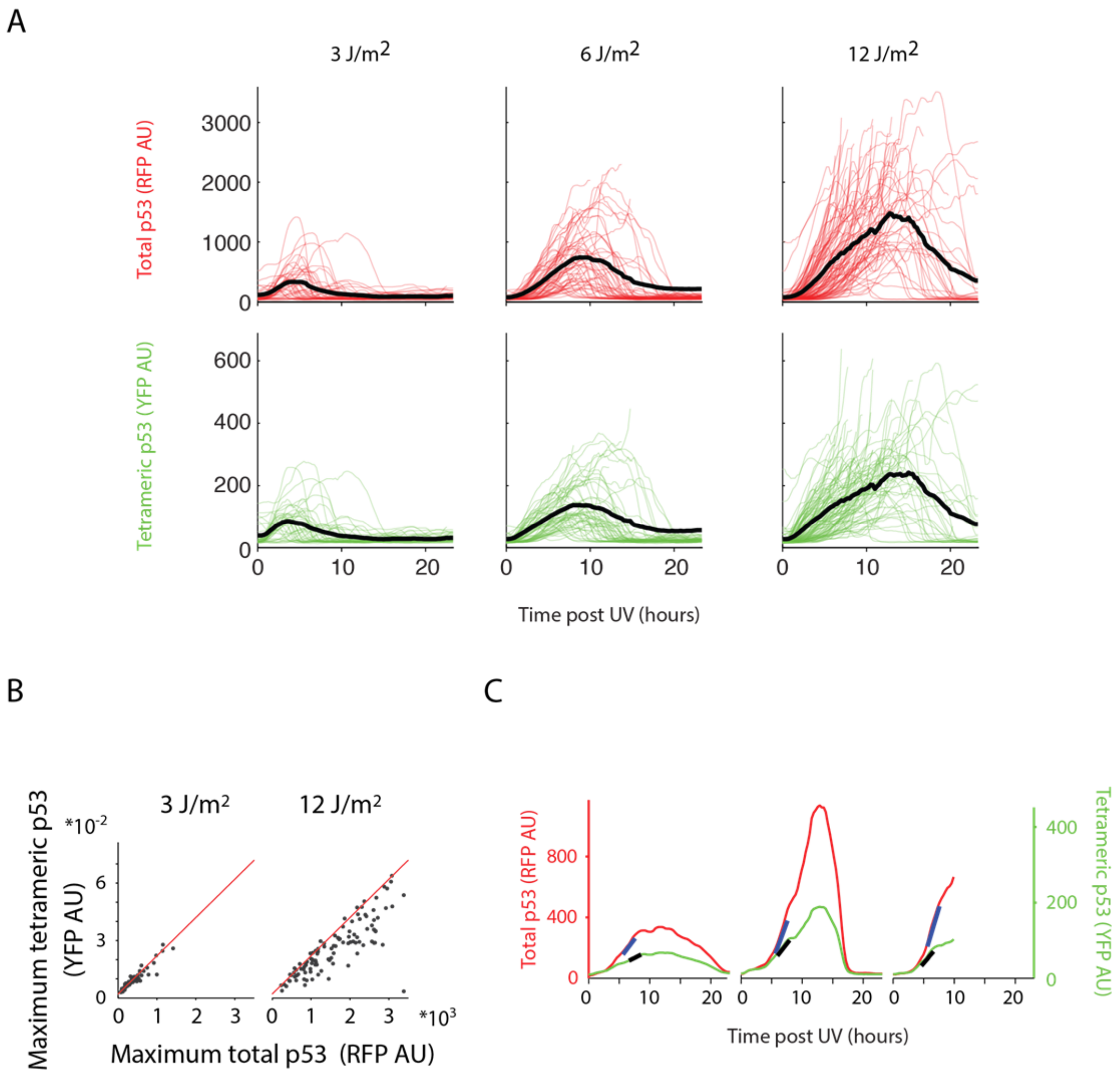

Supplementary Figure S3: Single cells trajectories and distributions of maximum total and tetrameric p53 and examples of slope calculation.

(**A**) Quantification of total and tetrameric p53 following 3, 6 and 12 J/m^2 UV. Each trace is a single cell. Black traces represent the mean dynamics (n = 100). (**B**) Scatter plot of the YFP peak versus the RFP peak after 3 and 12 J/m^2 UV. Each dot represents one cell. The red line represents the fit obtained from cells following 3 J/m^2, and is presented at 12 J/m^2 as a reference to compare the distributions between damage levels. At a low UV dose (3 J/m^2) the peak levels were linearly correlated. If tetramerization follows total p53 levels linearly, one would expect both the RFP and YFP signal to increase proportionally and to follow the red line under various UV doses. Instead, at a high UV dose (12 J/m^2) cells shifted to the right of the red line in the scatter plot, indicating that the increase in tetramerization does not match the increase in total p53. (**C**) Example of single cell traces of total p53 (RFP traces in red) and tetrameric p53 (YFP traces in green). The black and blue lines represent the values calculated for the slope of increase for each of the traces.

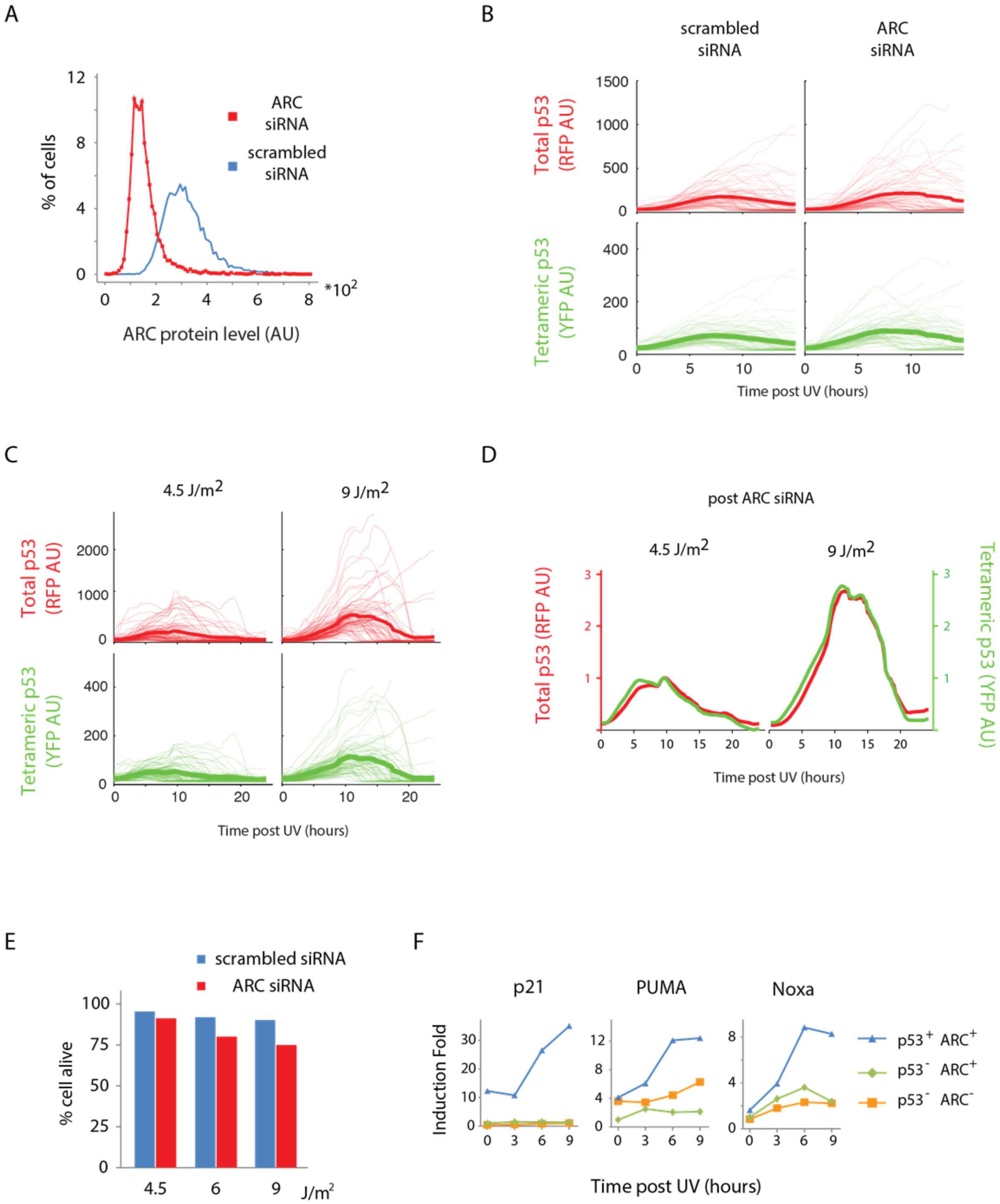
A
% of cells
12
8
4
0
0
2
4
6
8
*10^2
ARC protein level (AU)
ARC siRNA
scrambled siRNA
B
scrambled siRNA
ARC siRNA
Total p53 (RFP AU)
1500
1000
500
0
Tetrameric p53 (YFP AU)
400
200
0
0
5
10
Time post UV (hours)
C
4.5 J/m2
9 J/m2
Total p53 (RFP AU)
2000
1000
0
Tetrameric p53 (YFP AU)
400
200
0
0
10
20
Time post UV (hours)
D
post ARC siRNA
4.5 J/m2
9 J/m2
Total p53 (RFP AU)
Tetrameric p53 (YFP AU)
3
2
1
0
5
10
15
20
Time post UV (hours)
E
scrambled siRNA
ARC siRNA
% cell alive
100
75
50
25
0
4.5
6
9
J/m2
F
p21
PUMA
Noxa
Induction Fold
30
20
10
0
12
8
4
0
0
3
6
9
p53+ ARC+
p53- ARC+
p53- ARC-
Time post UV (hours)

Supplementary Figure S4: Supporting evidence of ARC knockdown.
(**A**) Distribution of nuclear ARC protein measured by immunofluorescence (n>5000). (**B**) Quantification of total (red) and tetrameric (green) p53 following 6 J/m^2 UV after scrambled siRNA control or ARC siRNA. Each trace is a single cell. Thick traces represent the mean dynamics (n = 100). (**C**) Dynamics of total and tetrameric p53 following siRNA against ARC measured by vYFP PCA after 4.5 J/m^2 and 9 J/m^2 UV. (**D**) Traces were normalized to the respective maximum level of 4.5 J/m^2 UV treatment, n = 180. The damping effect on p53 tetramerization is not observed in the absence of ARC. (**E**) qPCR of p53 target genes mRNA following 6 J/m^2 UV. $p53^-$ indicates cells carrying stable shRNA against p53; ARC^- indicates cells treated with siRNA against ARC. (**F**) Percentage of surviving cells determined by microscopy 12 hours post UV irradiation in cells treated with either scrambled control siRNA (blue bars) or ARC siRNA (red bars), n>300.

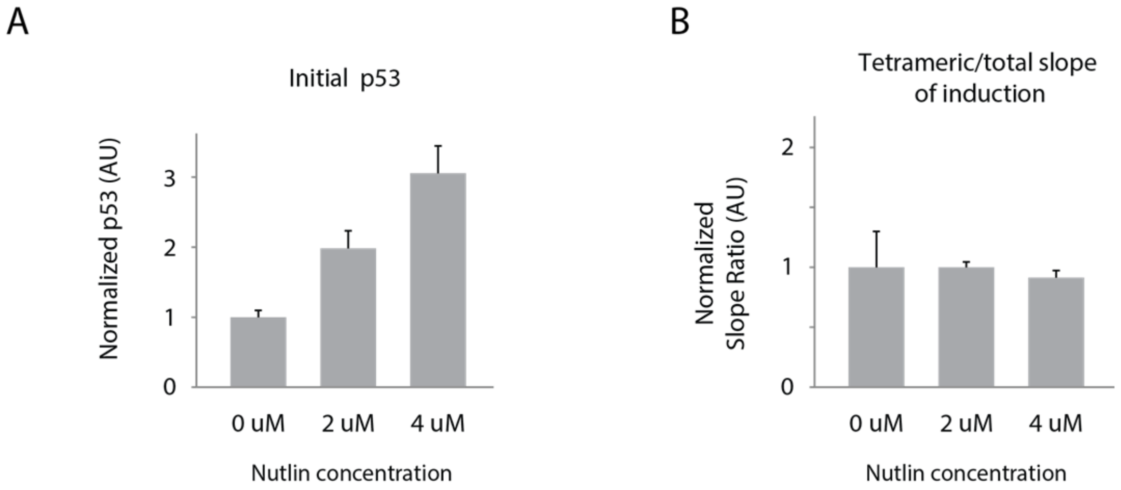

Supplementary Figure S5: The initial levels of p53 do not affect the rate of tetramerization
Cells were treated with Nutlin- 3 (Vassilev et al, 2004) 2 hours before UV irradiation of 6 J/m^2. Initial p53 levels (**A**) and rate of tetramerization (**B**) were measured using fluorescence microscopy. Even though we were able to increase the level of total p53 by up to 3 fold the rate of tetramerization does not change ($p>0.14$, K-S test). Bars graphs represent the median and SEM ($n>100$) and were normalized for the respective no Nutlin-3 condition.

Supplementary Table S1: description of source data for reproducing main text figures. Excel spreadsheets containing data and MatLab scripts used to produce Figure 2 and Figure 3 D-F are provided as source data. This table details the contents of the spreadsheets and the text files to recreate the figures using MatLab, MathWorks.

**File Name**	**Description**	**Sheet(s) Name**	**Notes**
Dataset 1.xlsx	Raw values of nuclear fluorescence to reproduce Fig 2B, 2C and S3A	TimeTraces UV xx RFP, TimeTraces UV xx YFP	Time = first column Cell ID = first row xx = 3, 6 and 12
Dataset 2.xlsx	Rise time values to reproduce Fig 2F	RiseTime UV xx RFP, RiseTime UV xx YFP	xx = 3, 6 and 12
Dataset 3.xlsx	Slope values to reproduce Fig 2G	Slope UV xx RFP, Slope UV xx YFP	xx = 3, 6 and 12
Dataset 4.xlsx	Values to reproduce Fig 3D	Max RFP, Max YFP, Slope RFP, Slope YFP	two columns = siCtrl, siARC
Dataset 5.xlsx	Values to reproduce Fig 3D	Slope YFP	Four columns = siARC UV4.5, siARC UV9, siCtrl 4UV.5, siCtrl UV9
plot figures 2BFG S3A.m	MatLab code to create figure 2BFG and S3A from Dataset 1-3		
plot figure 3DE.m	MatLab code to create figure 3DE from dataset 4		
plot figure 3F.m	MatLab code to create figure 3F from dataset 5		

SUPPLEMENTARY EXPERIMENTAL PROCEDURES

Co-Immunoprecipitation assay.

MCF7 p53shRNA cells were infected with lentivirus expressing pEF1a-p53wt-CFP and pEF1a-p53(wt/L344A/L344P)-HA and selected by antibiotic resistance. Cells were treated with 400 ng/µl neocarzinostatin (NCS, Sigma) and collected after 2.5 hrs. Cell lysate were obtained with lysis buffer without SDS (TNTE /1% Triton buffer supplemented with protease and phosphatase inhibitors) passing samples through 20G needle 10X at 4°C then spun down in chilled table top centrifuge for 30min. Lysates were incubated for 1 hour at 4°C in rotating shaker with monoclonal anti-HA-agarose beads (A2095, Sigma) pre-washed 3X in TNTE buffer. Lysate-beads solutions were washed 3X in TNTE buffer then resuspended in 4X LDS buffer and boiled at 95°C for 3 min.

Glutaraldehyde Lysate Crosslinking assay

After treatment and cell collection, cell lysates were obtained with lysis buffer without SDS supplemented with protease and phosphatase inhibitors and shear stress, then spun down in chilled table top centrifuge for 30min. Lysates were incubated for 30 minutes at 4°C with 0.025% or 0.075% final glutaraldehyde concentration. 4X LDS loading buffer and DTT 50uM were added and samples were boiled at 95°C for 3 min to stop fixation reaction.

Immunofluorescence

Cells were grown on coverslips coated with poly-L-lysine and fixed with 2% paraformaldehyde for 15 min at RT. Cells were permeabilized with 5 min incubation in 100% methanol at -20°C, washed in PBS, incubated with primary antibody against ARC (Cayman Chemical, 160737) , washed, and incubated with secondary antibody coupled to Alexa555. After washing, cells were stained with DAPI and embedded in Prolong Antifade (Invitrogen). Images were acquired with a 20x plan apo objective (NA 0.75) with the CY3 filter set (sp102v1, Chroma). Automated segmentation was performed in Matlab (MathWorks). 5,000-8,000 cells were measured per condition.

SUPPLEMENTARY REFERENCES

Vassilev LT, Vu BT, Graves B, Carvajal D, Podlaski F, Filipovic Z, Kong N, Kammlott U, Lukacs C, Klein C, Fotouhi N, Liu EA (2004) In vivo activation of the p53 pathway by small-molecule antagonists of MDM2. *Science* **303:** 844-848
