# Supplementary material for: Constant rate of p53 tetramerization in response to DNA damage controls the p53 response
Source: Mol Syst Biol. 2014 Oct 24;10(10):1–8. doi: 10.15252/msb.20145168 (PMC4299375; doi:10.15252/msb.20145168)
Supplement: Supplementary file 1 — Supplementary Information [file msb0010-0753-sd1.pdf]

Constant rate of p53 tetramerization in response to DNA damage controls the p53 response

Giorgio Gaglia and Galit Lahav

Corresponding author: Galit Lahav, Harvard Medical School

Review timeline:

Submission date:	29 January 2014
Editorial Decision:	25 February 2014
Revision received:	10 June 2014
Editorial Decision:	13 July 2014
Appeal:	25 July 2014
Editorial Decision:	20 August 2014
Revision received:	22 August 2014
Editorial Decision:	27 August 2014
Revision received:	04 September 2014
Accepted:	08 September 2014

Editor: Maria Polychronidou

Transaction Report:

(Note: With the exception of the correction of typographical or spelling errors that could be a source of ambiguity, letters and reports are not edited. The original formatting of letters and referee reports may not be reflected in this compilation.)

1st Editorial Decision 25 February 2014

Thank you again for submitting your work to Molecular Systems Biology. We have now heard back from the three referees who agreed to evaluate your manuscript. As you will see from the reports below, the referees acknowledge that you address a potentially interesting topic. However, they raise a series of concerns, which should be carefully addressed in a revision of the manuscript.

Overall, the referees think that as it stands, the study seems somewhat preliminary and point out that further experiments are required to robustly support the main conclusions. Without repeating all the points listed below, among the more fundamental issues are the following:
- Additional experimental evidence is required to demonstrate more convincingly that only p53 tetramerization leads to the formation of active YFP, while p53 dimerization does not.
- The potential effect of the split YFP in driving tetramerization needs to be excluded (point #2 of reviewer #2).
- The role of ARC in controlling the tetramerization of p53 should be examined in further detail. Reviewer #3 includes constructive suggestions in this regard.

If you feel you can satisfactorily deal with these points and those listed by the referees, you may wish to submit a revised version of your manuscript. Please attach a covering letter giving details of the way in which you have handled each of the points raised by the referees. A revised manuscript will be once again subject to review and you probably understand that we can give you no guarantee at this stage that the eventual outcome will be favorable.

© European Molecular Biology Organization

REFEREE REPORTS

Reviewer #1:

In the manuscript, the authors report that the formation of tetramers is the critical control point for p53 activation in response to UV-irradiation and that this control is performed by ARC. In principally, monitoring the level of tetramerization as an indication of p53 activation is a very interesting possibility and the proposed methodology is an elegant way for doing so. However, the manuscript has several flaws and drawbacks.

The first problem is the measurement of p53 tetramerization. The authors rely here on two mutations in the tetramerization domain of p53. The one mutation, L344P, shall not allow the formation of oligomers while the second mutation, L344A, shall allow the formation of dimers. To prove this principle, the authors used a) the immunofluorescence signal of proteins where p53 was fused to the red-fluorescent-protein as well as to one half of the yellow-fluorescent-protein (YFP). Upon, dimerization the two parts of YFP come to close proximity, resulting in a fluorescent signal (Figure 1E, F) and by b) co-immunoprecipitation of CFP- and HA-tagged p53 (Figure S1). The authors describe that with both p53 mutations, they did not get formation of heterodimers and they conclude that therefore the increase in fluorescence of the wt protein can only be due to tetramerization. The assays, that the authors perform are, however, not sufficiently convincing to discriminate between dimer and tetramer formation. First of all in figure 2, particularly in the online form, there is a shadow of a signal visible for L344A and if the authors would have developed the blot of the pull-down assay to the same intensity as the input control, this signal would surely be clearly visible also in the printed version. This result is then in contrast to Figure 1E,F, where there is no difference in intensity between the two different mutants. A stronger exposure of the pull-down of figure S1, to avoid the impression of "hiding something" and a Western blotting experiment after cross-linking to clearly show the amount of monomers, dimers and tetramers of the different constructs (e.g. like in Lomax et al., 1988) would certainly help to clarify this point.

The second and major problem is the poor description of the set up of some experiments. For example in figure S2, the authors show co-immunoprecipitation of biochemically tagged p53 where each dot shall represent a single cell. The authors did, however, not describe how they co-immunoprecipitated p53 from a single cell and monitored the level of co-immunoprecipitation at the single cell level. Likewise, on page 6/7, the authors write about the rise time and the slope for formation of p53 tetramers in response to UV-radiation. They, however, do not describe how they determined the rise time, how they determined the slope and how they distinguished between these two. Since this is a major read out of the paper, it is important to understand the methodology behind this investigation.

A third problem of the present study is that the third part is merely a conformation of the study by Foo et al., 2007. Instead of going too deeply in the description of the phenomenon, (which has already been done by Foo et al., 2007), it would have been better, if the authors would have gone beyond the findings of Foo et al., and explain the activation of p53 in response to UV-irradiation, its control by ARC and describe how this control influences the slope of p53 tetramer formation upon DNA damage.

Minor:
- It would be better if more of the graphs would be supported by microscope data (e.g. like in figure 1A and 1B). Some of them could e.g. be provided as supplementary data.
- In the material and methods section, the symbols are not properly presented.
- Figure 1H is not really helpful and could be removed.

Reviewer #2:

In this manuscript the authors want to examine whether p53 is regulated by a throttle mechanism. The term is borrowed from engineering and refers to a p53 inhibitor (referred to as tunable valve in

© European Molecular Biology Organization

this manuscript), whose activity increases as the intensity of the signal activating p53 also increases. The authors conclude that cells have a damping p53 activity at high UV levels.

My concerns are as follows:

1. The authors rely on a split YFP protein to monitor p53 tetramerization. One subunit of p53 is fused to the N-terminal half of YFP and another subunit to the C-terminal half of YFP. For reasons that are not clear to me and which contradict my experience with p53 oligomerization, both subunits of a p53 dimer contain the same half of YFP. Thus, p53 dimerization does not lead to formation of active YFP. However, when two dimers of p53 interact, then they bring together different halves of YFP, leading to YFP fluorescence.

To accept this, I need to see experimental evidence. If the authors co-express FLAG-tagged full-length p53 L344A and HA-tagged full-length p53 L344A in cells, then they should not be able to detect heterodimers containing both the FLAG and HA tags. Even better, instead of the L344A mutant, which is problematic, they should use the Arrowsmith dimerization mutant that has two amino acid substitutions and which forms better dimers than the L344A mutant. As a control, they should use full-length p53 with a wild-type tetramerization domain. Heterodimerization can be detected by co-immunoprecipitation.

The authors provide a co-immunoprecipitation along these lines and claim that heterodimers do not form. However, I see a faint band in Suppl. Fig. 1. Further, the C-terminus of p53 has regulatory function and fusing proteins or tags to the C-terminus can compromise its oligomerization properties (as we have found from experience). Have the authors tried to fuse their tags or fluorescent proteins to the N-terminus of p53?

Along the same lines, what is the evidence that the p53-L344A-RFP-YFP proteins form dimers in cells? Could they be mostly monomeric, which would be entirely consistent with the data presented by the authors in Fig. 1 and Suppl. Fig. 1? My recollection of the literature is that a large fraction of the L344A mutant is monomeric.

2. Assuming that the authors are right about L344A being a homodimer in cells, I would like to see some evidence that the split YFP does not itself affect tetramerization. My experience with split YFP proteins is that they drive oligomerization (especially, in this case, in which there are 2 N-terminal halves and 2 C-terminal halves). Also how does the fluorophore maturation kinetics affect the tetramerization reporter? Is there a way to show that the YFP signal is proportional to the tetramer concentration in the cell (or at least in vitro using purified proteins and measuring tetramerization by biophysical methods and then correlating the biophysical measurements to YFP fluorescence)? If possible, place all RFP-YFP reporters, tags, etc at the N-terminus of p53.

3. Ionizing radiation is a better way to activate p53. p53 responds poorly to UV and UV has much stronger non-specific effects in cells, as compared to IR.

4. The magnitude of the effect of ARC siRNA on the slope of p53 tetramer is small (Fig. 3F). The effect of ARC siRNA on p21 transcription could be explained by an effect of ARC on apoptosis. If ARC-depleted cells are less likely to undergo apoptosis, then one observes higher p21 levels. This has been observed previously in the p53 field. For example, weakly active p53 mutants seem to have higher transcriptional activity than wild-type p53, because the latter kills the transfected cells. Can the authors monitor apoptosis of the cells in this experiment? Does ARC depletion affect the activities of other transcription factors? (to show that the effect of ARC is specific for p53)

Reviewer #3:

Gaglia and Lahav report on the investigation of the dynamics of the formation of active p53 tetramers and compare this dynamics to the dynamics of the production of total p53. This is a very interesting and important topic and the methods chosen should be able to address this question. However, the study seems a bit too preliminary for publication and the technical details are not well presented.
A major question that has to be addressed is how the authors calculate the slope. At which point do

they determine the slope of the curve. I do not understand how the authors derive relatively small error bars for the slope (for example figure 2G) when the raw data shown in figure 2B show huge differences. Based on the data in figure 2B I would estimate that the error of the slope should be as high as the values themselves.
What are the units of the slope in figure 2G? What are the arrows marked with a and b pointing at in figure 2 A?
The authors show that using siRNA against the protein ARC increases the rate of formation of tetramers. This looks quite convincing and the explanation that ARC inhibits the formation of tetramers seems to make sense. However, this model is only valid if p53 is not vastly more abundant in the cell than ARC. What are the approximate ratios of both proteins? A prediction from this model would also be that not only silencing of ARC would lead to an acceleration of the Formation of tetramers but also the overexpression of p53 at concentrations much higher than the concentration of ARC. This could be shown with transiently transfected cells and increasing the plasmid concentration. ARC is thought to bind to the oligomerization domain, disrupting the formation of tetramers and exposing a nuclear export signal. Since the authors are experts in fluorescence microscopy and have fluorescently tagged p53 it would be very easy to investigate this question. Overall, while the topic is very interesting, further, crucial control experiments to support the model of ARC being responsible for this effect are missing and a better documentation of the quantitative analysis would be important.

Minor points: The Headline for Supplementary Figure 1 and 2 are the same. Why do the authors in Sup Fig 1 use a drug to damage DNA and not UV irradiation like in all other experiments? It is also not clear to me what is shown in Sup Fig. 3 A.

1st Revision - authors' response 10 June 2014

Response to comments highlighted by the Editor:

1) Additional experimental evidence is required to demonstrate more convincingly that only p53 tetramerization leads to the formation of active YFP, while p53 dimerization does not.
We now include additional experimental support for this claim using another dimer mutant, p53 M340E L344K (Davison et al, JMB, 2001), as was specifically suggested by reviewer #2. We repeated 2 essential control experiments with this mutant. First, we tagged the mutant p53 M340E L344K with HA and repeated the co-immunoprecipitation experiment with p53-CFP. We obtained similar results as the ones obtained with the original dimer mutant L344A. Specifically, both dimer mutants led to a very faint band of p53-CFP even after a long exposure of the membrane (**new Supplementary Figure 1D**). This very weak band implies that a small fraction of p53 dimers might be heterodimers. This can potentially lead to a fluorescence signal of the YFP split system. However when we tagged the new dimer mutant p53 M340E L344K with the BiFC reporter system we saw that the ratio of YFP to RFP is similar to the ratio obtained with the monomeric mutant p53 L344P (**new Supplementary Figure 1A**). The fact that we see no increase in the YFP/RFP signal with both dimer mutants (Figure 1F & Supplementary Figure 1A**)** convinced us that the minimal levels of heterodimers, if exist, do not affect the fluorescence signal and that our system is suitable for quantifying p53 tetramers. We added these new results to the Supplementary Information and openly discuss their implications and significances.

2) The potential effect of the split YFP in driving tetramerization needs to be excluded (point #2 of reviewer #2).
This is an important point. As suggested by Reviewer #2, we performed a lysate glutaraldehyde crosslinking experiment (as described in Foo et al, PNAS 2007) on cell lines expressing either wild-type or dimeric mutant p53 L344A tagged with the split YFP system. We observed a tetrameric band only with wild-type p53 but not with the dimer mutant, confirming that the split YFP does not induce spurious tetramerization (**New Figure 1H**). In addition, we showed that the dynamics of total p53 is similar between cells carrying both fragments of the split YFP and cells carrying only one of the two fragments (**new Supplementary Figure 2**). p53 dynamics in response to DSBs were previously shown to depend on p53 degradation and transcriptional activity (Batchelor et al, Mol Cell, 2008), both affected by p53 tetramerization (Gaglia et al 2013). The fact that the split YFP system does not alter these well characterized dynamics argues

© European Molecular Biology Organization

that tetramerization is not affected by the reporter system.

3) The role of ARC in controlling the tetramerization of p53 should be examined in further detail. Reviewer #3 includes constructive suggestions in this regard.
Following reviewer's #3 comments we increased the concentration of p53 prior to UV and quantified the rate of tetramerization. We found that a 3-fold increase in initial p53 levels does not affect the rate of tetramerization (**new Supplementary Figure 5**). We discussed alternative explanations for this result, including a competitive model in which ARC abundance is much higher than p53, or a transient binding between ARC and p53 resulting in disruption of tetramers. Distinguishing between these or other potential models will require a large effort including the use and development of other experimental tools beyond the scope of our study.

Response to additional comments raised by the reviewers:

Reviewer #1:

1) The assays, that the authors perform are, however, not sufficiently convincing to discriminate between dimer and tetramer formation. First of all in figure 2, particularly in the online form, there is a shadow of a signal visible for L344A and if the authors would have developed the blot of the pull-down assay to the same intensity as the input control, this signal would surely be clearly visible also in the printed version.
This result is then in contrast to Figure 1E,F, where there is no difference in intensity between the two different mutants. A stronger exposure of the pull-down of figure S1, to avoid the impression of "hiding something" and a Western blotting experiment after cross-linking to clearly show the amount of monomers, dimers and tetramers of the different constructs (e.g. like in Lomax et al., 1988) would certainly help to clarify this point.
As discussed in response to the editor's comment #1 above, we addressed this concern using another dimerization mutant, p53 M340E L344K, in both imaging and pull-down experiments with a longer exposure of the membrane (4 minutes; wild-type p53 shows a saturated signal after 10 seconds of exposure; **new Supplementary Figure 1D**). The faint bands observed with the two dimer mutants indeed raise the possibility that a very small fraction of dimers are heterodimers. However, since no additional fluorescence signal was observed with two distinct dimeric mutants (Figure 1F and **new Supplementary Figure 1A**) we concluded that such potential small level of hetero-dimers are insignificant for affecting the fluorescence signal obtained by our imaging system which therefore reports on tetramers. Note that the pull-down assay could capture indirect interactions between p53 molecules, not driven by the tetramerization domain but by other binding partners. The BiFC assay is hence more specific to local and direct interactions. We now openly discuss these results and the differences in sensitivity between the two experimental methods in the text.
As suggested by the reviewers we also performed a crosslinking experiment on p53 wild-type and L344A (**new Figure 1H**), and showed that tetramers are formed only by the wild-type form of p53. The dimer mutant p53 L344A formed dimers efficiently as was previously reported (Mateu et al. NatStructBio 1999, Gaglia et al. PNAS 2013).

2) The second and major problem is the poor description of the set up of some experiments.
- For example in figure S2, the authors show co-immunoprecipitation of biochemically tagged p53 where each dot shall represent a single cell. The authors did, however, not describe how they co-immunoprecipitated p53 from a single cell and monitored the level of coimmunoprecipitation at the single cell level.
- Likewise, on page 6/7, the authors write about the rise time and the slope for formation of p53 tetramers in response to UV-radiation. They, however, do not describe how they determined the rise time, how they determined the slope and how they distinguished between these two. Since this is a major read out of the paper, it is important to understand the methodology behind this investigation.
Co-immunoprecipitation was performed at the population level and not in single cells. Supplementary Figure 2 (now Supplementary Figure 3) and the Materials and Methods sections were changed to clarify this confusion and to include more detailed explanations of our experimental set-ups and analyses.

3) A third problem of the present study is that the third part is merely a conformation of the study by Foo et al., 2007. Instead of going too deeply in the description of the phenomenon, (which has already been done by Foo et al., 2007), it would have been better, if the authors would have gone beyond the findings of Foo et al., and explain the activation of p53 in response to UVirradiation, its control by ARC and describe how this control influences the slope of p53 tetramer formation upon DNA damage.

Indeed Foo et al. 2007 were the first to identify ARC as an inhibitor of p53 tetramerization and we mentioned and cited their work in our manuscript. It is important to note that Foo et al investigated p53 tetramers mainly in unperturbed conditions, without temporal resolution and mostly at cell populations level. Our study focuses on the dynamics of activation of p53 tetramerization after DNA damage in single cells. In this context we relied on the previous knowledge gained from Foo et al and quantitatively determine the effect of ARC on specific dynamical features of p53 activation following UV.

In addition, as mentioned in our response to the editor comment #3, we now show that different initial levels of p53 do not increase the rate at which tetramers are formed (**new Supplementary Figure 5**) and we discuss potential models that can explain these results.

Minor:

- It would be better if more of the graphs would be supported by microscope data (e.g. like in figure 1A and 1B). Some of them could e.g. be provided as supplementary data.

We provided examples of the microscope data (images) in Figure 1E and Figure 2A. We do not think that including additional images will be instructive, since it is very difficult to gain quantitative understanding from the raw images. We can include such additional images if the reviewer insists and the editor finds it helpful.

- In the material and methods section, the symbols are not properly presented.

In our submitted pdf the symbols were presented well. We apologize if the reviewer received a version in which symbols were presented poorly and will make sure this is not the case when the paper is published.

- Figure 1H is not really helpful and could be removed.

We removed Figure 2H, which we believe is the one the reviewer was referring to (there was no Figure 1H).

Reviewer #2:

1.1 The authors rely on a split YFP protein to monitor p53 tetramerization. One subunit of p53 is fused to the N-terminal half of YFP and another subunit to the C-terminal half of YFP. For reasons that are not clear to me and which contradict my experience with p53 oligomerization, both subunits of a p53 dimer contain the same half of YFP. Thus, p53 dimerization does not lead to formation of active YFP. However, when two dimers of p53 interact, then they bring together different halves of YFP, leading to YFP fluorescence.

To accept this, I need to see experimental evidence. If the authors co-express FLAG-tagged full-length p53 L344A and HA-tagged full-length p53 L344A in cells, then they should not be able to detect heterodimers containing both the FLAG and HA tags.

Even better, instead of the L344A mutant, which is problematic, they should use the Arrowsmith dimerization mutant that has two amino acid substitutions and which forms better dimers than the L344A mutant. As a control, they should use full-length p53 with a wild-type tetramerization domain. Heterodimerization can be detected by co-immunoprecipitation.

As suggested by the reviewer and discussed in response to the editor's comment #1, we repeated the co-immunoprecipitation with one of the Arrowsmiths dimeric double mutants (p53 M340E L344K; Davison TS et al, JMB, 2001) and obtained similar results as with the L344A mutant **(new Supplementary Figure 1C, D**).

1.2 The authors provide a co-immunoprecipitation along these lines and claim that heterodimers do not form. However, I see a faint band in Suppl. Fig. 1.

See our response to Editor comment #1 and reviewer 1, comment #1.

1.3 Further, the C-terminus of p53 has regulatory function and fusing proteins or tags to the Cterminus can compromise its oligomerization properties (as we have found from experience).

Have the authors tried to fuse their tags or fluorescent proteins to the N-terminus of p53?
The C-terminal fusion of p53 has been previously well characterized by us and others and was shown to mimic the dynamics of endogenous p53 (Lahav et al 2004, Batchelor et al 2008, Loewer et al. 2010, Gaglia et al. 2013), suggesting that it does not alter the regulation on p53 oligomerization. Nevertheless, we tried an N-terminal fusion of p53 as suggested by the reviewer and found that it significantly alters the regulation and dynamics of p53 (Figure 1 below)

Revision Figure 1

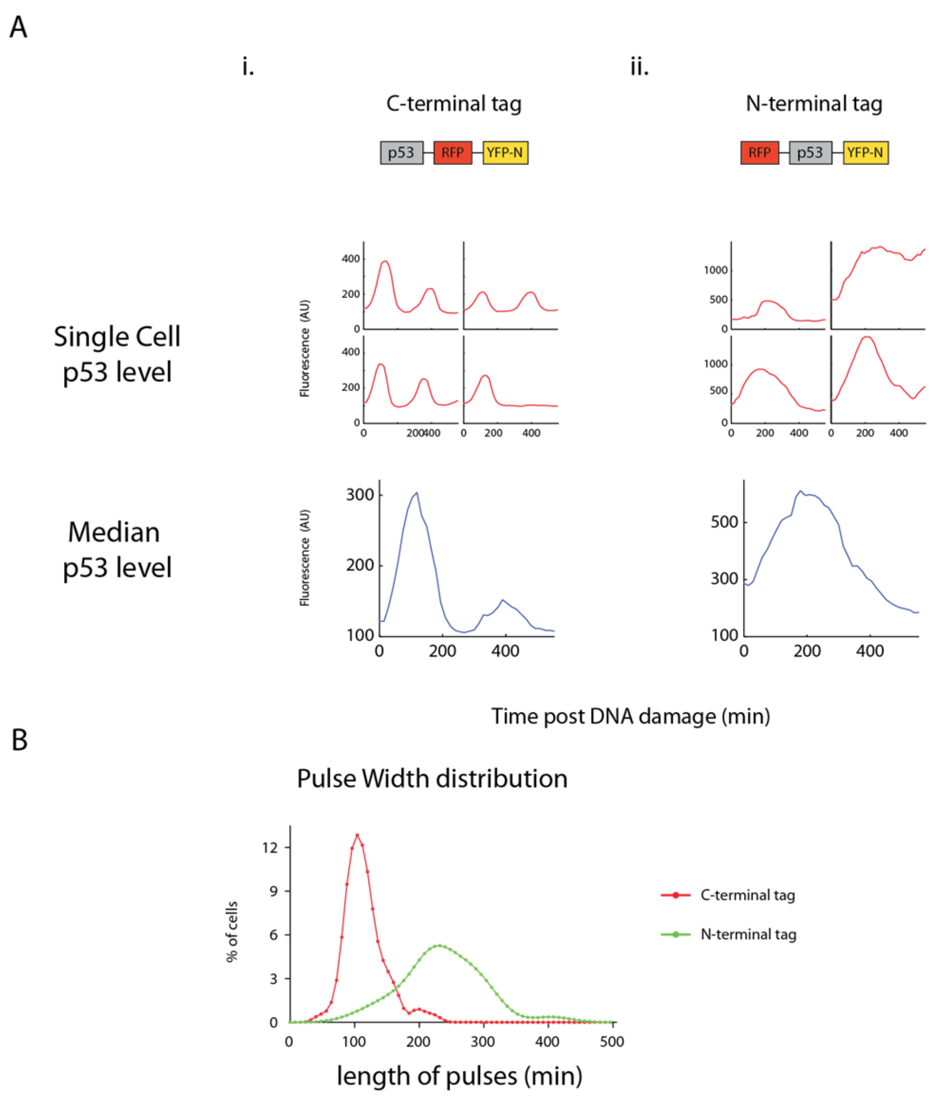

Revision Figure 1: N-terminal fluorescence tag disrupts p53 dynamics.
(A) MCF7 cells were transformed with one of the BiFC construct in which RFP is either fused to the C-terminus (i) or N-terminus (ii) of p53. The dynamics of total p53 reported by the RFP signal after double strands breaks induced by NCS (400ng/mL) are presented at single cell level and at median population level (n>40). The C-terminus tag shows dynamics that are similar to the previously well-characterized dynamics with reporters and endogenous p53. The N-terminal tag significantly alters p53 dynamics. (B) Quantification of the width of total p53 for C- and N-terminal tag measured by the full-width half-maximum (p < 10-5).

1.4 Along the same lines, what is the evidence that the p53-L344A-RFP-YFP proteins form dimers in cells? Could they be mostly monomeric, which would be entirely consistent with the

© European Molecular Biology Organization

data presented by the authors in Fig. 1 and Suppl. Fig. 1? My recollection of the literature is that a large fraction of the L344A mutant is monomeric.
This is an important point. We have previously shown that the p53 L344A mutant forms dimers in cells (Gaglia G et al, PNAS 2013). We now clearly mention this in the text. In addition, we now include new cross-linking results showing the formation of dimers with the p53 L344A mutant (**new Figure 1H**).

2. Assuming that the authors are right about L344A being a homodimer in cells, I would like to see some evidence that the split YFP does not itself affect tetramerization. My experience with split YFP proteins is that they drive oligomerization (especially, in this case, in which there are 2 N-terminal halves and 2 C-terminal halves). Also how does the fluorophore maturation kinetics affect the tetramerization reporter? Is there a way to show that the YFP signal is proportional to the tetramer concentration in the cell (or at least in vitro using purified proteins and measuring tetramerization by biophysical methods and then correlating the biophysical measurements to YFP fluorescence)? If possible, place all RFP-YFP reporters, tags, etc at the N-terminus of p53.
Related to split-YFP potentially driving tetramerization - see our response to editor comment #2. We agree that maturation kinetics can be an issue when comparing different fluorophores. However, in our analysis we consistently compared data from the same fluorophore, across different doses of UV damage. We chose not to take the *in-vitro* route since we believe that our new cross-linking data, together with the new co-immunoprecipitation and imaging results are sufficient for arguing that the YFP signal reports on p53 tetramerization. Lastly, as mentioned in response to comment 1.3, N-terminal fusion of p53 significantly disrupts its dynamics.

3. Ionizing radiation is a better way to activate p53. p53 responds poorly to UV and UV has much stronger non-specific effects in cells, as compared to IR.
In our system p53 strongly responds to UV. We intentionally chose to focus on this type of damage since it was previously shown to have a dose-dependent effect on p53 levels (Batchelor et al. 2011) as oppose to ionizing radiation that triggers p53 pulses with fixed amplitude and duration across a wide range of IR doses (Lahav et al 2004).

4. The magnitude of the effect of ARC siRNA on the slope of p53 tetramer is small (Fig. 3F). The effect of ARC siRNA on p21 transcription could be explained by an effect of ARC on apoptosis. If ARC-depleted cells are less likely to undergo apoptosis, then one observes higher p21 levels. This has been observed previously in the p53 field. For example, weakly active p53 mutants seem to have higher transcriptional activity than wild-type p53, because the latter kills the transfected cells. Can the authors monitor apoptosis of the cells in this experiment? Does ARC depletion affect the activities of other transcription factors? (to show that the effect of ARC is specific for p53)
This is an interesting point. ARC is considered to be a repressor of apoptosis. ARC siRNA therefore should increase the likelihood for cells to undergo apoptosis. As suggested by the reviewer we quantified the number of apoptotic cells in control and ARC siRNA treated cells in response to UV and observed a small increase in apoptosis (12hr post treatment; **new Supplementary Figure 4D**). This may result from the induction of p53 target genes Puma and Noxa, in ARC siRNA cells as presented in Figure 3G or by other apoptotic pathways regulated by ARC. We have not looked at the global effect of ARC on other transcription factors, as this will require a major effort which is outside the scope of our work. We did show that the induction of p21, PUMA and NOXA in ARC siRNA is p53-dependent (Supplementary Figure 4C).

Reviewer #3:

1. A major question that has to be addressed is how the authors calculate the slope. At which point do they determine the slope of the curve. I do not understand how the authors derive relatively small error bars for the slope (for example figure 2G) when the raw data shown in figure 2B show huge differences. Based on the data in figure 2B I would estimate that the error of the slope should be as high as the values themselves.
What are the units of the slope in figure 2G? What are the arrows marked with a and b pointing at in figure 2 A?
We clarified these points in the Material and Methods section of the paper, adding more details

© European Molecular Biology Organization

in the Figure legends and modifying Figure 2A and 2G. We also added examples of how our algorithm performed in calculating the slope on a few sample traces (**new Supplementary Figure 3B**).

2. The authors show that using siRNA against the protein ARC increases the rate of formation of tetramers. This looks quite convincing and the explanation that ARC inhibits the formation of tetramers seems to make sense. However, this model is only valid if p53 is not vastly more abundant in the cell than ARC. What are the approximate ratios of both proteins? A prediction from this model would also be that not only silencing of ARC would lead to an acceleration of the Formation of tetramers but also the overexpression of p53 at concentrations much higher than the concentration of ARC. This could be shown with transiently transfected cells and increasing the plasmid concentration.
We thank the reviewer for the constructive comments, which led us to investigate the effect of initial p53 levels on the rate of tetramerization. See our response to the editor comment #3.

3. ARC is thought to bind to the oligomerization domain, disrupting the formation of tetramers and exposing a nuclear export signal. Since the authors are experts in fluorescence microscopy and have fluorescently tagged p53 it would be very easy to investigate this question.
In our experimental system endogenous p53 and the fluorescently tagged p53 are strongly nuclear (Figure 1E, Figure 2A and Loewer et al Cell 2010). Our system is therefore limited in its ability to detect a potential reduction in the cytoplasmic fraction of p53 following ARC siRNA.

4. Minor points: The Headline for Supplementary Figure 1 and 2 are the same. Why do the authors in Sup Fig 1 use a drug to damage DNA and not UV irradiation like in all other experiments? It is also not clear to me what is shown in Sup Fig. 3 A.
We changed the title of the figure to match the content and clarified the legend of the supplementary figure pointed out by the reviewer.
p53 tetramerization was previously characterized and quantified in response NCS using other single cell methods (Gaglia et al PNAS 2013). In order to establish the efficacy and test the accuracy of the BiFC reporter, we decided in Supplementary Figure 1 to use similar experimental conditions.
We clarified the legend of the figure the reviewers is referring to, now Supplementary Figure 4A.

2nd Editorial Decision 13 July 2014

Thank you again for submitting your revised work to Molecular Systems Biology. We have now heard back from the two referees who agreed to evaluate your manuscript. As you will see from the reports below, the referees still raise significant concerns on your work, which, I am afraid to say, preclude its publication in Molecular Systems Biology.

In particular, there are significant issues regarding the reliability of the presented assay for measuring p53 activity. The high 'constitutive' YFP signal observed with monomeric/dimeric p53 (Fig. 1F) results in an overall low signal-noise ratio for the assay (< 2 standard deviations from the data shown in Fig. 1F). The background YFP signal does not seem to be taken into account in the subsequent experiments/quantifications, in which the YFP signal is considered as a measure of tetrameric p53. Therefore, as it stands, it remains unclear whether the reported conclusions on the dynamics of tetrameric p53 formation, are supported.

An additional important point raised by both reviewers is that the proposed mechanism for the control of p53 tetramerization by ARC is insufficiently supported.

Moreover, the differential behavior of the C-terminally tagged p53 and the N-terminally tagged version is concerning. The construct described as N-terminally tagged in Revision Figure 1A (ii), seems somewhat puzzling in the sense that it is N-terminally tagged with RFP but at the same time it is also tagged C-terminally with YFP-N. As such, the comparison of the two constructs seems inconclusive.

We realize that the additional points 2 and 3 raised by reviewer #2 reflect misunderstandings and are addressable.

© European Molecular Biology Organization

Overall, we appreciate the elegant principle underlying the proposed assay for quantifying p53 activity in single cells. However, since the major findings are based on this single assay, the issues raised on its reliability undermine the robustness of the conclusions, while the mechanistic insights provided by the study remain preliminary.

Considering the overall rather low level of support provided by the referees and the fact that our editorial policy allows in principle a single round of major revision, I see no other choice than to return the manuscript with the message that we cannot offer to publish it. In any case, thank you for the opportunity to examine your work. I hope that the points raised in the reports will prove useful to you and that you will not be discouraged from submitting future work to Molecular Systems Biology.

REFEREE REPORTS

Reviewer #2:

I remain unconvinced by this study for the following reasons:

1. Fig. 1E and 1F. It seems that the YFP/RFP ratio in the L344A and L344P mutants is quite high compared to the same ratio for wild-type p53 (about 70% of the wild-type signal). But the authors interpret their findings, as if the YFP signal for the monomeric and dimeric p53 (L344A and L344P) was zero. The high background of monomeric p53 YFP fluorescence makes it hard to measure accurately the effect of tetramerization.

2. The whole idea of the throttle mechanism is that total p53 levels may increase, but the levels of active p53 would increase less. In Fig. 2A, the cells with high RFP signal (total p53) also have high YFP signal (active p53). I therefore fail to see dampening of the levels of active p53 as proposed by the authors.

3. Similarly, Fig. 2C shows proportionally similar increases in total and active p53 levels following different doses of UV (6 and 12 J/m2). Where is the dampening?

4. As mentioned before the effects of ARC siRNA on the slope (Fig. 3F) are of a very small magnitude.

5. The different behavior of the N-terminally and C-terminally tagged p53 molecules (Fig. 1 in the rebuttal) is worrying. Why do we trust the C-terminally tagged p53, but not the N-terminally tagged p53? My experience with p53 indicates that N-terminal tags are usually well-tolerated.

6. The whole system employed by the authors to measure p53 activity is problematic. The assay relies on large tags (RFP plus a YFP fragment, which total about 300 amino acids, almost as large as p53 itself; interaction between YFP fragments that are denatured on their own; a very high background (70% of full activity); and an indirect assay of YFP fluorescence, versus a more direct assay of activity, such as DNA binding or transcription.

Reviewer #3:

The authors have addressed most of the issues raised. However, I am not convinced that ARC is the only factor that acts as a buffer for dimeric p53. Otherwise the overexpression experiments of p53 should have yielded a different result. The speculation that ARC is much higher in concentration than p53 might be right but unfortunately no evidence is given and I doubt that with overexpressing p53 will be the case. Proteins of the S100 protein family are also known to affect the tetramerization equilibrium and the possibility that other proteins than ARC also influence the formation of tetramers should at least be included in the discussion.

© European Molecular Biology Organization

Appeal 25 July 2014

Thank you for your response. I can certainly understand why you decided to reject the paper based on reviewer 2's negative comments. However, this review contains misinterpretations, which should be of concern. I would appreciate it very much if you would look closely at what reviewer 2 has said in light of what he/she had said previously. I outline below some of the misunderstandings by reviewer 2 so that you will have some context for my concerns.

Given the novel approach we describe here, the importance of the findings, and given reviewer 3's comment that most of the issues have been addressed, we ask that you reconsider your decision or ask for advice from a third reviewer.

Examples of reviewer 2's comments demonstrating misunderstanding of the manuscript that has led to wrong conclusions and low enthusiasm:

1. Fig. 1E and 1F. It seems that the YFP/RFP ratio in the L344A and L344P mutants is quite high compared to the same ratio for wild-type p53 (about 70% of the wild-type signal). But the authors interpret their findings, as if the YFP signal for the monomeric and dimeric p53 (L344A and L344P) was zero. The high background of monomeric p53 YFP fluorescence makes it hard to measure accurately the effect of tetramerization.

We disagree. The difference between wild-type p53 and the mutants, while small, is measurable, significant and reproducible. Each measurement represents at least 50 cells, and the p values are statistically significant (p < 10-14, p53wt versus p53L344A; p < 10-17 p53wt versus p53L344A).

Note that, in Figure 2B & C we intentionally chose to present the raw data, in the most open honest way and without subtracting the background. As I am sure you could appreciate, subtracting the background would have led to a larger effect. Our presentation therefore is more conservative and honest and we are sorry that the reviewer failed to see that. In addition, in the follow up figures we were careful to analyze and present our results in a way that is not affected by the background. This included comparisons between UV doses (Fig. 2D), calculating rise times and slopes (Fig. 2F, G), factors that are independent of the background. Our main conclusion about the damping effect was based on these analyses.

2 +3. The whole idea of the throttle mechanism is that total p53 levels may increase, but the levels of active p53 would increase less. In Fig. 2A, the cells with high RFP signal (total p53) also have high YFP signal (active p53). I therefore fail to see dampening of the levels of active p53 as proposed by the authors. Similarly, Fig. 2C shows proportionally similar increases in total and active p53 levels following different doses of UV (6 and 12 J/m2). Where is the dampening?

As you have noted, these statements are incorrect and represent misunderstanding.

4. As mentioned before the effects of ARC siRNA on the slope (Fig. 3F) are of a very small magnitude.

Small magnitude doesn't mean "not real" or "not important". The "very small magnitude" effect of ARC siRNA (Fig. 3F) is significant, reproducible and leads to a large effect on p53 transcriptional function (Fig. 3G). It therefore represents real biology.

It is of a concern to only trust biological measurements that show high magnitude. Many important inputs lead to a small change in magnitude, yet are very critical for cells. It is obviously challenging to measure changes of small magnitudes; it requires careful analysis, many repeats and more controls; all of which were included in this work.

5. The different behavior of the N-terminally and C-terminally tagged p53 molecules (Fig. 1 in the rebuttal) is worrying. Why do we trust the C-terminally tagged p53, but not the N-terminally tagged p53? My experience with p53 indicates that N-terminal tags are usually well-tolerated.

We disagree. We have more than 10 years of experience with fluorescently tagged p53. We fully trust the C-terminal tagged p53, since we have extensively shown that it faithfully represents the

dynamics of endogenous p53 and does not alter its transcriptional activity (see references 1-6 at the end). While we can appreciate that other proteins might better tolerate N-terminal tags, this is not the case for p53 based also on work from other world-leading p53 laboratories that have used C-terminal tagged p53 (see references 7-12 at the end).

6. The whole system employed by the authors to measure p53 activity is problematic. The assay relies on large tags (RFP plus a YFP fragment, which total about 300 amino acids, almost as large as p53 itself; interaction between YFP fragments that are denatured on their own; a very high background (70% of full activity); and an indirect assay of YFP fluorescence, versus a more direct assay of activity, such as DNA binding or transcription.

Large tags can potentially affect protein activity, however as discussed above, we have previously shown in great details that this is not the case for C-terminally fluorescently tagged p53.
The activity of p53 in single cells can in principle be quantified using a transcriptional reporter, in which a target gene promoter drives the expression of a fluorescent protein. However, in the p53 pathway different target genes show different patterns of activation, implying that their induction depends on additional factors beyond p53 and making it impossible (and wrong) to choose a single promoter as a general readout for p53 activity. We deliberately chose tetramerization as a valuable measure for the functional unit of p53 as it has been shown to be fundamental for its ability to bind DNA and activate transcription globally. We will be happy to add the motivation behind our approach to the paper.

REFERENCES

1. Lahav, G., Rosenfeld, N., Sigal, A., Geva-Zatorsky, N., Levine, A. J., Elowitz, M. B., and Alon, U. (2004) Nat Genet 36, 147-150
2. Batchelor, E., Mock, C. S., Bhan, I., Loewer, A., and Lahav, G. (2008) Mol Cell 30, 277-289
3. Loewer, A., Batchelor, E., Gaglia, G., and Lahav, G. (2010) Cell 142, 89-100
4. Batchelor, E., Loewer, A., Mock, C., and Lahav, G. (2011) Mol Syst Biol 7, 488
5. Purvis, J. E., Karhohs, K. W., Mock, C., Batchelor, E., Loewer, A., and Lahav, G. (2012) Science 336, 1440-1444
6. Gaglia, G., Guan, Y., Shah, J. V., and Lahav, G. (2013) Proc Natl Acad Sci U S A 110, 15497-15501
7. Boyd, S. D., Tsai, K. Y., and Jacks, T. (2000) Nat Cell Biol 2, 563-568
8. Li, M., Brooks, C. L., Wu-Baer, F., Chen, D., Baer, R., and Gu, W. (2003) Science 302, 1972-1975
9. Geva-Zatorsky, N., Rosenfeld, N., Itzkovitz, S., Milo, R., Sigal, A., Dekel, E., Yarnitzky, T., Liron, Y., Polak, P., Lahav, G., and Alon, U. (2006) Mol Syst Biol 2, 2006 0033
10. Toledo, F., Liu, C. W., Lee, C. J., and Wahl, G. M. (2006) Nucleic Acids Res 34, e92
11. Demidenko, Z. N., Korotchkina, L. G., Gudkov, A. V., and Blagosklonny, M. V. (2010) Proc Natl Acad Sci U S A 107, 9660-9664
12. Gogna, R., Madan, E., Kuppusamy, P., and Pati, U. (2012) J Biol Chem 287, 2907-2914

3rd Editorial Decision 20 August 2014

After discussing your manuscript once again and considering the points mentioned in your letter and during our phone conversation, we have consulted an additional reviewer (#4), who was specifically asked to evaluate the points raised by reviewer #2 on the reliability of the presented assay for measuring p53 activity.

In summary, reviewer #4 thinks that the assay is reliable for detecting p53 tetramers <i>in vivo</i>. According to this reviewer, the potential effect of the fluorescent reporter on p53 turnover is a more important issue compared to the signal to background ratio of the assay. Since based on the results shown in Supplementary Figure S2 there do not appear to be any serious effects on p53 turnover, the assay seems to be reliable. Additionally, reviewer #4 includes some suggestions regarding the description of the assay and the related reagents and recommends using the term 'Protein-fragment complementation assay' (PCA) instead of BiFC.

Taken together, we would like to ask you to address the points below and those raised by referee #4, in a revision of this work. In particular:
- The statistical significance of the differences in the slope of p53 tetramerization should be reported, taking into account the sensitivity and variability of the assay.
- We would suggest including a depiction of the distribution of single cells' trajectories and the mean dynamics of total vs tetrameric p53 (similar as in Figure 2B), in addition to the bar charts in panels 2F, 2G, 3E, 3F in order to better visualize the data.
- The advantages of the proposed approach compared to i.e. the use of transcriptional reporters as suggested by reviewer #2 should be briefly mentioned.

2nd Revision - authors' response 22 August 2014

Editorial comments:

1. *The statistical significance of the differences in the slope of p53 tetramerization should be reported, taking into account the sensitivity and variability of the assay.*
We added the statistical significances of the differences (p-values < 0.05) to Figures 1F, 2D, 2G, 3D-F, S1A, S5B; and we wrote the p-values in the corresponding legends.

2. *We would suggest including a depiction of the distribution of single cells' trajectories and the mean dynamics of total vs tetrameric p53 (similar as in Figure 2B), in addition to the bar charts in panels 2F, 2G, 3E, 3F in order to better visualize the data.*
Figure 2C already presented the mean single cell dynamical data that is analyzed in 2F and 2G. We now added the single cell trajectories and mean dynamics of total and tetrameric p53 corresponding to Figure 2F, G (Supplementary Figure 3) and to Figure 3E, F (Supplementary Figure 4).

3. *The advantages of the proposed approach compared to i.e. the use of transcriptional reporters as suggested by reviewer #2 should be briefly mentioned.*

We added to the introduction (page 4) a paragraph describing the limitations of other approaches for measuring the activity of p53's transcriptional activity (by localization or the use of transcriptional reporters) and the advantage of our approach.

Reviewer #4 comments:

So in conclusion, I believe the assay can be used to detect tetramers with reasonable accuracy in vivo. At any rate, I do not believe that any other comparable assay would do better. Again, it is not so much the signal to background of the assay that is important here, but the effects of the PCA components on p53 turnover. Since there are no serious effects, I think that the assay works to its purpose.
We thank the reviewer for looking so carefully at our study and acknowledging the reliability and accuracy of our assay.

1) *I object to the terminology BiFC as it obscures the intellectual contributions of the inventors of the technique. Many authors, like those of this manuscript, mistakenly cite Hu and Kerrpolla, Mol. Cell, 2002 as the reference of the assay. The contribution of that paper was implementation of the practically identical assay previously described in E. coli, but in mammalian cells. The assay was invented in Lynn Regan's group at Yale as described in:*
Ghosh, I., et al. (2000). "Antiparallel leucine zipper-directed protein reassembly: Application to the green fluorescent protein." Journal of the American Chemical Society122(23): 5658-5659.
We thank the reviewer for bringing this to our attention. We corrected the text and added the appropriate reference from Regan's group.

2) *Hu and Kerrpolla coined the expression BiFC, ignoring an established literature that referred to similar assays based on different reporter enzymes as Protein-fragment complementation Assays (PCA). The history and conceptual basis for this general approach to measure protein-protein interactions was described in:*

© European Molecular Biology Organization

Michnick, S. W., et al. (2007). "Universal strategies in research and drug discovery based on protein-fragment complementation assays." Nat Rev Drug Discov 6(7): 569-582.
We changed the term BiFC to PCA throughout the manuscript and also cited this and additional related papers.

3) *The authors obtained the PCA components they used from the Michnick lab, for a specific variant of the GFP based on the Venus variant of YFP, but they failed to cite the paper where this is described. They should replace BiFC throughout the manuscript with Venus YFP PCA, which they could shorten to vYFP PCA. The correct citation for this PCA is:*
Remy, I., et al. (2004). "PKB/Akt modulates TGF-beta signalling through a direct interaction with Smad3." Nat Cell Biol 6(4): 358-365.
We changed the terminology as suggested by the reviewer and added the reference describing the PCA components.

4) *The figure (1B) and description of the PCA are wrong. PCA results from refolding of the enzyme reporter from fragments, not binding of pre-folded fragments as the figure implies. The correct representation and description of PCA principles is described in the above reference (Michnick, et al., 2007). Also, the fragments are not, as the authors describe, "halves" of the YFP polypeptide. A full description of how the protein was dissected is described in Ghosh, et al, 2000.*
We changed Figure 1B, G and the related text to reflect the fact that the fragments are not pre-folded before binding. We also changed the word “halves” to “fragments”.

5) *Finally, unlike other PCAs, those based on fluorescent proteins are irreversible. Once the reporter protein refolds, it cannot unfold even if the proteins that brought them together dissociate. The authors need to discuss this issue and why it does not affect their ability to use the PCA for their purposes. They should read and cite:*
Magliery, T. J., et al. (2005). "Detecting protein-protein interactions with a green fluorescent protein fragment reassembly trap: scope and mechanism." J Am Chem Soc127(1): 146-157.
We now specifically mention that in our PCA assay the binding between fragments is irreversible. We discuss the potential effect of such binding on p53 regulation and connect it with the data showing no effect of the PCA fragments on p53 dynamics (Supplementary Figure 2). We also cite the reference suggested by the reviewer.

4th Editorial Decision 27 August 2014

Thank you again submitting your revised manuscript to Molecular Systems Biology. We are now satisfied with the modifications made and we think that the main concerns of the referees have been addressed.

Prior to formal acceptance of your manuscript we would like to encourage you to include the source data for the figure panels that show essential quantitative results.

3rd Revision - authors' response 04 September 2014

We have made the changes according to your suggestions and MSB’s Author guidelines.
